# Experience with health care integration in Bidasoa integrated health organisation: development of the continuity of care unit/Experiencia de integración en la Organización Sanitaria Integrada Bidasoa: desarrollo de la Unidad de Continuidad Asistencial

**Published:** 2012-05-29

**Authors:** Iñaki Berraondo Zabalegui

**Affiliations:** Managing Director, Bidasoa Integrated Health Organisation, Hondarribia, Basque Country, Spain

**Keywords:** continuity of patient care, continuidad asistencial

## Introduction

Bidasoa integrated health organisation (IHO) was established in January 2011, bringing together the hospital and the three health centres of the Bidasoa health region. A new organisational model was introduced to integrate the resources of the region in order to facilitate:

- A change in the model for the relationships between professionals; and
- A change in the model for the provision of care.

## Description of the project

### A. Changing the model for the relationship between professionals:

To improve collaboration we have focused on the following areas:

1. Creation of culture-cultural change:
  - Design and communication of the Strategic Plan
  - Communication between units and professionals
  - Creation of a joint technical committee as a body for clinician participation
  - Creation of clinical and health care joint committees (primary care and hospital): patient safety, pharmacy, clinical records, etc.
  - Setting up of a shared Intranet
2. Integration of the clinical system:
  - Progress towards the introduction of a shared medical record
  - Development of integrated care pathways (ICPs): care pathways that specify the relationships between professionals participating in the provision of care related to a specific health problem. In 2011, the ICPs for atrial fibrillation, heart failure (HF) and chronic obstructive pulmonary disease (COPD) were designed.
  - Development of clinical guidelines
  - Establishment of a continuity of care unit (CCU) composed of an internist, a liaison nurse and a social worker.
3. The aims of the CCU are:
  - to support primary care (PC), providing one-stop appointments
  - to provide a health care unit for patients with multiple conditions as described below.

The referral internist (one for each health centre) is responsible for the admission of patients with complex or multiple conditions in the event that they require admission to hospital. The mission of the CCU is to stabilise patients and facilitate continuity of care by the PC doctor and nurse. These patients have in place a continuity of care plan (CCP) between levels of care. The role of the liaison nurse is to support the patient in his/her transition from hospital to home, where they are followed up by PC.

The referral internist visits the health centre every other week to undertake clinical sessions with the PC professionals, and is available to general practitioners at any time for any queries they may have.

1. Management and leadership:
  - Development of shared leadership with the setting up of clinical management units in the three health centres
  - Establishment of a transparency information system.

### Change in the healthcare model

Bidasoa IHO has adopted a healthcare strategy in line with the Kaiser Permanente model, defining actions for the various levels of the care pyramid:

- For complex cases, we have started to identify patients with high levels of comorbidity
- For patients with a lower level of complexity (middle level of the Kaiser pyramid), continuity of care is provided with PC through the work of the liaison nurse and the ICPs for HF and COPD
- With regards to the lower level of the Kaiser pyramid, Bidasoa IHO involves an intervention for patients with recent-onset diabetes, as identified using the adjusted clinical group (ACG) system.

We describe the activity of the CCU in more detail below.

## Results

In th first five months of operation of the CCU, we identified 57 complex patients, according to the definition of Ollero et al. [1], with an average of 2.5 admissions and 3.2 attendances to the Emergency Department in that period. In 2010, they had an average of 2.6 admissions and 4.3 emergency attendances. These first data indicate that the first patients benefiting from the unit are individuals with a rapid deterioration but also show some positive trends. The number of visits to the Emergency Department of the first 30 patients (recruited in the beginning of the period) has decreased and continuity of care has been established that allows changes in their state of health to be detected immediately, facilitating programmed admissions (it should be taken into account that 24 out of the 54 patients were recruited in the last two months of this five-month period).

The data collected on the first 30 patients cared for under the CCP are the following:

- 70 admissions in 2010 (2.3 admissions per patient)
- 39 admissions in the first 9 months of 2011

That is, so far there has been a 25% decrease in the number of admissions. In this sample of patients, we also found that there were 112 attendances to Emergency Department in 2010, while in the first 9 months of 2011 there have been just 35 (a 58% decrease).

Nine months after the setting up of the CCU, 115 patients had been allocated to CCPs. The average age of patients was 79.5 years, they were on multiple medications (an average of 8 drugs), attended an average of 9 consultations with specialists and were classified as at high social risk (41%).

For the identification of complex patients, other sources of information have also been used:

- We used the Ambulatory Care Sensitive Conditions classification. A total of 73 patients were admitted to Bidasoa Hospital due to COPD. They had an average of 7 diagnoses at discharge from hospital, 23 of them having high blood pressure (HBP) and 11 diabetes mellitus (DM).
- Further, 135 patients were admitted due to heart failure in 2010. These patients had an average of 8.9 diagnoses at discharge, 63 patients having HBP and 43 DM.
- Finally, we analysed information from the ACGs. We identified 414 people aged 65 or below with a high level of comorbidity. Only 27% of these individuals were admitted to Bidasoa Hospital in 2010, which may suggest that they were seen at the tertiary level hospital. Further, we identified 53 people above 65 years of age with a high level of comorbidity of whom 45% were admitted to Bidasoa Hospital.

## Conclusions

The have modified the focus of the models for care and for the relationship between professionals. The creation of the CCU has resulted in a decrease in hospital admissions and in attendances to the Emergency Department by patients with multiple conditions.

Nearly half the patients that were admitted for HF and/or COPD are candidates for care under a CCP and the remaining are less complex chronic patients.

## Conference abstract Spanish

## Introducción

La Organización Sanitaria Integrada (OSI) Bidasoa se crea en enero de 2011 integrando los tres centros de salud de la comarca Bidasoa con el Hospital. Se estructura un modelo de organización diferente, para integrar los recursos de la región con el objetivo de facilitar:

- Un cambio en el modelo de relación entre profesionales y
- Un cambio en el modelo asistencial.

## Descripción de la experiencia:

### Cambio en el modelo de relación entre profesionales

Para avanzar en la colaboración se ha trabajado alrededor de:

1. Creación de cultura – cambio cultural:
  - Elaboración y comunicación del Plan Estratégico
  - Comunicación entre unidades y entre profesionales
  - Consejo Técnico mixto como órgano de participación asistencial.
  - Comités mixtos (atención primaria y hospital) clínico asistenciales: seguridad de pacientes, farmacia, documentación clínica, …
  - Intranet común …
2. Integración del sistema clínico:
  - Avance hacia la historia clínica compartida
  - Desarrollo de GPSs: rutas asistenciales que delimitan las relaciones entre los profesionales que participan en el proceso asistencial relacionado con un problema de salud concreto. En 2011, se han diseñado los GPS de fibrilación auricular, Insuficiencia Cardiaca y EPOC.
  - Desarrollo de vías clínicas
  - Creación de una Unidad de Continuidad Asistencial (UCA), que integra a tres internistas, una enfermera de enlace y una trabajadora social.
3. La UCA tiene como objetivos:
  - servir de apoyo al médico de atención primaria (AP), actuando como consulta de alta resolución.
  - servir de unidad asistencial a los pacientes pluripatológicos como se describe más adelante.
  El internista de referencia (uno para cada centro de salud), es responsable de los ingresos de los pacientes complejos/pluripatológicos cuando ingresan en el hospital. La misión de la UCA es estabilizar a estos pacientes y facilitar la continuidad asistencial por parte del médico y enfermera de AP. Estos pacientes tienen activado un Plan de Continuidad (PAC) entre los dos niveles. La enfermera de enlace tiene un papel conductor del paciente hacia su domicilio en donde es seguido por AP.
  El internista de referencia se desplaza al centro de salud con periodicidad quincenal para sesiones clínicas con los profesionales de AP y mantiene una accesibilidad permanente para los médicos de familia.
4. Gobierno y liderazgo:
  - Desarrollo del liderazgo compartido con el desarrollo de Unidades de Gestión Clínica en los tres centros de salud
  - Sistema de información transparente

### Cambio en el modelo asistencial

OSI Bidasoa adopta una estrategia asistencial en línea con el modelo de Kaiser, definiendo actuaciones para los diferentes estratos del complejo asistencial:

- Para casos complejos se ha iniciado la identificación de pacientes con comorbilidad elevada.
- Para pacientes con menor nivel de complejidad (parte media de la pirámide de Kaiser), se facilita su continuidad asistencial hacia AP por medio de la enfermera de enlace y seguimiento de los GPS de Insuficiencia cardiaca y EPOC.
- En cuanto a la parte baja de la pirámide Kaiser, OSI Bidasoa interviene en la población de diabéticos poco evolucionados, identificados por ACGs (Adjusted Clinical Groups).

A continuación se describe en más detalle la actividad de la UCA.

## Resultados

En los 5 primeros meses de funcionamiento de la UCA se identificaron, siguiendo la definición de Ollero et al. [1], 57 pacientes complejos que han tenido en ese periodo un promedio de 2,5 ingresos y 3,2 visitas por urgencias. En 2010 tuvieron un promedio de 2,6 ingresos y 4,3 visitas en urgencias del Hospital. Estos primeros datos indican que los primeros pacientes que se han identificado son pacientes con un deterioro muy rápido pero ofrecen también un primer dato positivo. El número de visitas por urgencias de los 30 pacientes captados al inicio ha disminuido y se ha establecido una continuidad asistencial que permite detectar cambios en su estado de salud de forma inmediata, facilitándose ingresos programados (debe tenerse en cuenta que 24 de estos 54 pacientes se captaron en los dos últimos meses).

Para los 30 primeros pacientes incluidos en el PAC, se aprecian los siguientes datos:

- 70 ingresos es el año 2010 (2,3 ingresos por paciente)
- 39 ingresos en los primeros 9 meses de 2011

Es decir, una disminución de un 25% en el número de ingresos.

En estos pacientes se observa además que si en 2010 tuvieron 112 visitas por urgencias, en los nueve primeros meses de 2011 han necesitado acudir a urgencias en 35 ocasiones (disminución del 58%).

Nueve meses después del inicio de actividad de la UCA se han incluido en PACs a 115 pacientes. Pacientes que tienen una edad media de 79,5 años, polimedicados (8 fármacos de promedio), con 9 consultas de promedio con las especialidades. Riesgo social elevado (41%).

En el proceso de identificación de pacientes complejos, se han explorado también otras fuentes de información.

- Uso de los Ambulatory Care Sensivity Conditions: En el Hospital Bidasoa ingresaron 73 pacientes por EPOC. Estos pacientes tienen 7 Diagnósticos al alta, 23 tienen además Hipertensión Arterial (HTA) y 11 Diabetes Mellitus (DM). Ingresaron también 135 pacientes por Insuficiencia Cardiaca (IC) en 2010. Estos pacientes tienen 8,9 Diagnósticos al alta, 63 tienen además HTA y 43 DM.
- Finalmente, se ha explorado la información a partir de los ACGs. Se han identificado 414 personas menores de 65 años con elevado nivel de comorbilidad. Sólo el 27% ingresaron en el Hospital Bidasoa en 2010, lo que probablemente indica que son pacientes atendidos en el nivel terciario. También se han identificado 53 personas mayores de 65 años con elevado nivel de comorbilidad de las cuales ingresaron en el Hospital Bidasoa el 45%.

## Conclusiones

Se ha modificado el enfoque del modelo asistencial y el modelo de relación entre profesionales. La creación de la UCA ha resultado en una disminución de los ingresos hospitalarios y las visitas a urgencias de pacientes pluripatológicos.

La mitad de los pacientes que ingresan por IC y/o EPOC son susceptibles de ser incluidos en un PAC y la otra mitad aproximadamente pertenecerían a otro estrato menos complejo de la población de pacientes crónicos.
